# Visualizing spatial distribution of alectinib in murine brain using quantitative mass spectrometry imaging

**DOI:** 10.1038/srep23749

**Published:** 2016-03-30

**Authors:** Hiroaki Aikawa, Mitsuhiro Hayashi, Shoraku Ryu, Makiko Yamashita, Naoto Ohtsuka, Masanobu Nishidate, Yasuhiro Fujiwara, Akinobu Hamada

**Affiliations:** 1 Division of Clinical Pharmacology and Translational Research, Exploratory Oncology Research and Clinical Trial Center, National Cancer Center, 5-1-1 Tsukiji, Chuo-ku, Tokyo 104-0045, Japan; 2 Department of Molecular Imaging and Pharmacokinetics, National Cancer Center Research Institute, 5-1-1 Tsukiji, Chuo-ku, Tokyo 104-0045, Japan; 3 Shimadzu Techno-Research Inc., 3-19-2, Minamirokugo, Ohta-ku, Tokyo 144-0045, Japan; 4 Translational Clinical Research Science & Strategy Dept., Chugai Pharmaceutical Co., Ltd., 200 Kajiwara, Kamakura, Kanagawa 247-8530, Japan; 5 Department of Medical Oncology and Translational Research, Graduate school of Medical Sciences, Kumamoto University, 1-1-1 Honjo, Chuo-ku, Kumamoto 860-8556, Japan; 6 Strategic Planning Bureau, National Cancer Center, 5-1-1 Tsukiji, Chuo-ku, Tokyo 104-0045, Japan

## Abstract

In the development of anticancer drugs, drug concentration measurements in the target tissue have been thought to be crucial for predicting drug efficacy and safety. Liquid chromatography-tandem mass spectrometry (LC-MS/MS) is commonly used for determination of average drug concentrations; however, complete loss of spatial information in the target tissue occurs. Mass spectrometry imaging (MSI) has been recently applied as an innovative tool for detection of molecular distribution of pharmacological agents in heterogeneous targets. This study examined the intra-brain transitivity of alectinib, a novel anaplastic lymphoma kinase inhibitor, using a combination of matrix-assisted laser desorption ionization–MSI and LC-MS/MS techniques. We first analyzed the pharmacokinetic profiles in FVB mice and then examined the effect of the multidrug resistance protein-1 (MDR1) using *Mdr1a/b* knockout mice including quantitative distribution of alectinib in the brain. While no differences were observed between the mice for the plasma alectinib concentrations, diffuse alectinib distributions were found in the brain of the *Mdr1a/b* knockout versus FVB mice. These results indicate the potential for using quantitative MSI for clarifying drug distribution in the brain on a microscopic level, in addition to suggesting a possible use in designing studies for anticancer drug development and translational research.

During the drug development process, determination of whether a new compound can reach its intended target or not is one of the key factors for obtaining the proof of mechanism and concept^1^. A previous study has shown that plasma drug concentrations do not accurately predict the exposure in some organs and tissues^2^. To determine drug exposure in the target tissues, today, homogenized tissues are now commonly used for quantitative assessments by liquid chromatography tandem mass spectrometry (LC-MS/MS).

Although LC-MS/MS can accurately determine the average levels of a compound in a target organ or tissue, there is a disadvantage when using this technique, as there is a complete loss of the spatial information for the compound in the target tissue. When estimating the efficacy and safety of a compound, particularly for anti-tumor drugs, it is very important to know both the specific location of the compound and the concentration that is present at these points within the target tissue. In order to understand the complex biochemical interactions that occur between a drug and its target tissue, it is necessary to detect not only the level of exposure, but also the spatial distribution within the target tissue. Thus, the use of only LC-MS/MS analysis is insufficient for clarifying the behavior of compounds such as anti-tumor drugs^3^.

Matrix-assisted laser desorption ionization mass spectrometry imaging (MALDI-MSI) is one of the newer innovative technologies that are highly sensitive and can provide mass spectrum information in two dimensions. This technology makes it possible to measure the distribution of diverse molecular species in a tissue section, including endogenous molecules at a specific time, without destroying the target tissue or requiring the use of target-specific molecular labeling reagents^2^,^4^,^5^,^6^,^7^,^8^,^9^. Even though this technique has proved to be beneficial, some challenges remain regarding the quantitative capability and reproducibility.

Assessment of the drug concentration and distribution is not only of interest in tumors but also in the brain as well. The blood-brain barrier (BBB), in particular the multidrug resistance protein 1 (MDR1, also known as P-glycoprotein or ATP-binding cassette, sub-family B, member 1: ABCB1) has a central role in the drug distribution and transition to the brain parenchyma^10^. MDR1 is an important transporter protein located in the cell membrane that pumps many endogenous substances out of the cells. Moreover, this protein is highly expressed in various tissues, including the endothelial cells of the brain blood vessels and the intestinal epithelium^10^. While MDR1 has been shown to regulate the intestinal absorption of a wide variety of drugs, it has been frequently reported that the blood concentration of a drug does not necessarily correlate with the MDR1-determined substance properties^11^,^12^,^13^,^14^,^15^,^16^. For instance, a pharmacokinetics (PK) study showed that after administration of the anti-cancer drug, docetaxel, in FVB (wild-type, WT) and the *Mdr1a/b* knockout (KO) mice, there was no difference in the plasma concentrations. However, the brain concentrations in the *Mdr1a/b* KO mice were much higher than that observed in the FVB mice^15^.

In our current study, we employed a quantitative MSI (qMSI) method in a preclinical mouse model to evaluate the intra-brain distribution of a new anti-cancer drug, the echinoderm microtubule-associated protein-like 4 and anaplastic lymphoma kinase (EML4-ALK) inhibitor, alectinib^17^,^18^,^19^, which was approved for use in Japan on July 4, 2014. Using serial sections, we used the combination of MALDI-MSI and LC-MS/MS techniques to facilitate inter-sample comparisons and improve reproducibility of the analysis, in addition to investigating the effect of MDR1 on the intra brain transitivity of alectinib in *Mdr1a/b* KO mice. The aim of this study was to examine the potential of utilizing qMSI to translate drug delivery evaluations for use in new drug development and practical applications.

## Results

### Pharmacokinetics of alectinib in mice

**Figure 1:**
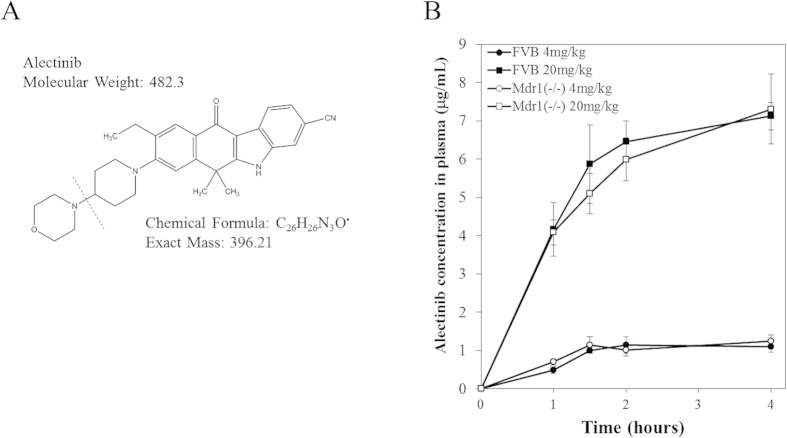
The plasma concentration of alectinib following oral administration in mice (FVB and *Mdr1a/b* (−/−)). (**A**) Chemical structure of alectinib. MW: 482.3. For the LC-MS/MS analysis, the main fragment, m/z 483.2 to 396.0, was chosen for quantitative purposes. (**B**) Plasma concentrations of alectinib in FVB mice (4 mg/kg dose: filled circle, 20 mg/kg dose: filled squire) and in *Mdr1a/b* (−/−) knockout mice (4 mg/kg dose: open circle, 20 mg/kg dose: open square). The plasma concentrations were measured by LC-MS/MS at 1, 1.5, 2, and 4 hours after an oral administration of alectinib. Data shown are the mean ± SD, n = 4.

Recently, there has been an increased interest in the pharmacokinetics of anti-tumor drugs in the brain from an efficacy and toxicity viewpoint^20^,^21^. Therefore, our current study focused on alectinib, which was recently approved for the treatment of lung cancer in Japan (Fig. 1A). We performed a pharmacokinetic analysis of alectinib in FVB and *Mdr1a/b* KO mice; Plasma, cerebrospinal fluid (CSF), and brain tissue were collected 1, 1.5, 2, and 4 hours after a single oral administration of a 4 and 20 mg/kg dose. In mice, a 20 mg/kg administration of alectinib resulted in higher plasma concentrations as compared to a 4 mg/kg administration (Supplemental Table 1). There were no significant differences in the alectinib plasma concentrations observed between the FVB and *Mdr1a/b* KO mice at each dosage (Fig. 1B).

The majority of the alectinib concentrations in the CSF were lower than the limits of quantitation (4 mg/kg group: 16/16 samples in FVB mice, 15/16 samples in *Mdr1a/b* KO mice; 20 mg/kg group: 9/16 samples in FVB mice, 7/16 samples in *Mdr1a/b* KO mice). In the samples that could be evaluated, there was no apparent correlation observed (Supplemental Table 2).

### Use of qMSI to visualize alectinib distribution in the mouse brain

**Figure 2:**
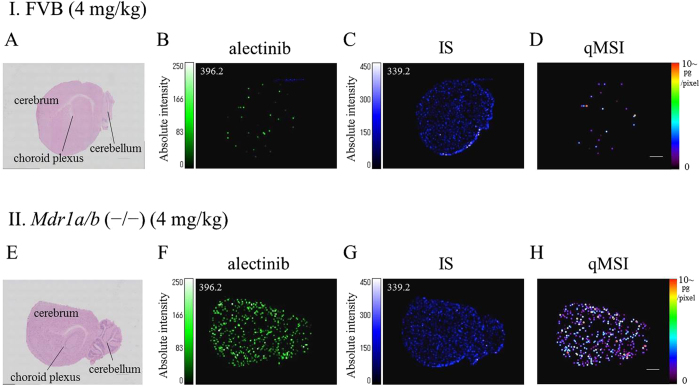
Alectinib distribution in the mouse brain at 4 hours after administration of a 4 mg/kg dose. (**A**,**E**) HE staining of sagittal brain sections from FVB and *Mdr1a/b* (−/−) mice, respectively. (**B**,**F**) Molecular images of alectinib. (**C**,**G**) Molecular images of internal standard (IS). (**D**,**H**) qMSI images of alectinib. MSI data were acquired at a spatial resolution of 80 μm in the positive ion mode, and the optimal transition of alectinib used for the MALDI-MSI was m/z 483.3 to 396.2. In the qMSI images of alectinib, ion intensity was converted into the amount of alectinib by using LC-MS/MS analysis of serial sections.

**Figure 3:**
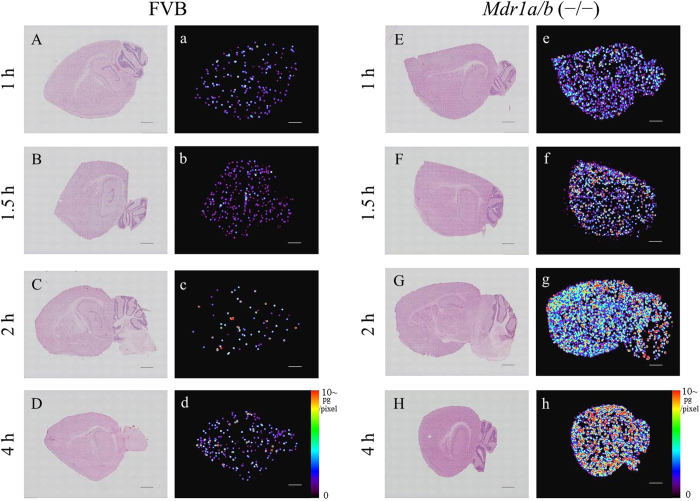
Comparison of the qMSI images of alectinib at each time point after administration of a 20 mg/kg dose. (**A**–**H**) HE staining and (a–h) qMSI images of alectinib of the brain tissue sections at 1, 1.5, 2, and 4 hours after oral administration of a 20 mg/kg dose in FVB and *Mdr1a/b* (−/−) mice, respectively. MALDI-MSI was performed with a spatial resolution of 80 μm. Scale bar: 1000 μm.

To determine alectinib transitivity into the mouse brain, we performed qMSI analysis (*Methods* and Supplemental Figure 1) using FVB and *Mdr1a/b* KO murine brain sections. Brain tissue collected included the cerebrum, cerebellum, and choroid plexus (Fig. 2). Alectinib qMSI was performed on mouse brains that were collected at 1, 1.5, 2 and 4 hours after the administration of alectinib (4 mg/kg, 20 mg/kg). There was a higher level of drug accumulation and a greater diffusion in the brain tissue section after the administration of 4 mg/kg of alectinib in the *Mdr1a/b* KO versus the FVB group (Fig. 2) (Supplemental Figure 2). After administration of 20 mg/kg of alectinib, there was a significantly increased alectinib exposure and diffused distribution of alectinib in *Mdr1a/b* KO versus FVB murine brains (Fig. 3).

**Figure 4:**
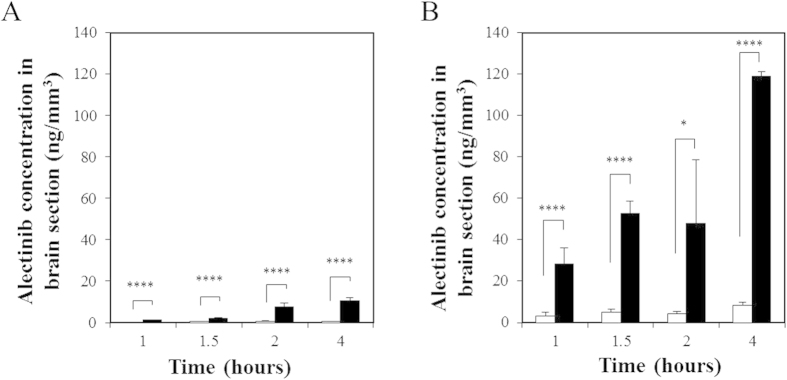
Quantification of alectinib in brain tissue sections by LC-MS/MS. Alectinib concentrations (ng/mm^3^) in brain tissue sections after a dose of 4 mg/kg (**A**) and 20 mg/kg (**B**) were measured by LC-MS/MS. FVB: open bars, *Mdr1a/b* KO: filled bars. Data shown are the mean ± SD. Statistically significant differences: ^*^P < 0.05 and ^****^P < 0.0005.

LC-MS/MS quantitation using serial sections of qMSI showed no obvious differences in section size among the samples (Supplemental Table 3) and indicated significantly higher average alectinib concentrations (per unit of section) in the *Mdr1a/b* KO murine brain versus the FVB brain under all conditions examined (Fig. 4) (Supplemental Figure 3). At 4 hours after administration of the 4 mg/kg (Fig. 4A) and the 20 mg/kg (Fig. 4B) doses, we observed an approximately a 20-fold (10.64 ± 1.58 vs. 0.53 ± 0.16 ng/mm^3^, *P* < 0.0001; unilateral *t*-test) and 14-fold (119.08 ± 2.31 vs. 8.24 ± 1.63 ng/mm^3^, *P* < 0.0001; unilateral *t*-test) accumulation of alectinib in the *Mdr1a/b* KO murine brains versus the FVB brains, respectively (Supplemental Table 4).

Quantitative distribution of alectinib shown by the qMSI was confirmed by using laser microdissection (LMD) of additional serial sections (Supplemental Figure 4). The calculated concentrations of the arbitrary regions R1 to R8 from the images of qMSI correlated well with the concentration of the corresponding LMD R1 to R8 regions measured by LC-MS/MS (Supplemental Table 5).

### Localization of alectinib in the brain blood vessels

**Figure 5:**
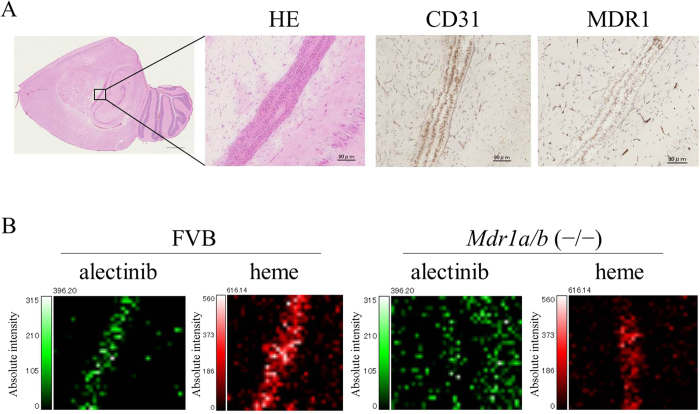
Localization of alectinib and blood vessels in FVB and Mdr1a/b knockout mouse brains. Both MSI and immunohistochemical (IHC) staining were used to determine the location of alectinib and blood vessels in the brains of FVB mice. (**A**) HE and IHC staining with anti-CD31 and anti-MDR1 antibody. (**B**) MALDI-MSI image of alectinib and heme in the area including the choroid plexus at a resolution of 20 μm at 4 hours after administration of the 20 mg/kg dose. The slices used for HE and IHC staining were the same serial sections examined during MSI analysis in the FVB brain.

The final step of the experiment focused on the histological intra brain localization of alectinib. After treating the FVB and *Mdr1a/b* KO mice with 20 mg/kg of alectinib, we used MSI at a 20-micrometer resolution and immunohistochemistry (IHC) to examine the localization of alectinib in the brain blood vessels (Fig. 5). In the FVB mice, MSI showed that the distribution of alectinib was mostly co-localized with the heme that was derived from the red blood cells (Fig. 5B), while IHC demonstrated there was distribution in the CD31 and MDR1 (Fig. 5A), which indicated the location of the blood vessels in the choroid plexus. On the other hand, a diffuse distribution of alectinib was observed in the *Mdr1a/b* KO mouse brain (Fig. 5B). By using MSI, it was possible to visualize the true distribution of alectinib in the brains of both the *Mdr1a/b* KO and the FVB mice. Thus, we were able to evaluate the precise pharmacokinetics of the drug in target tissues or organs at the micro level.

## Discussion

In this study, we propose application of this quantitative MSI method, which would add the quantitative capability to conventional MSI by using concurrent LC-MS/MS analysis of serial sections, into the visualizing study of spatial distribution of pharmaceutical agent in the brain. We evaluated the intra-brain distribution of alectinib and the effect of MDR1 (p-glycoprotein) on pharmaceutical profiles with MSI concurrently with a general LC-MS/MS approach by using an *in vivo* murine model. The alectinib PK study using *Mdr1a/b* KO mice showed that while the alectinib brain distribution was affected by MDR1, it was not associated with the blood concentration.

MSI can directly detect diverse molecular species on tissue sections simultaneously and utilize this information to create a drug distribution map without the use of target-specific molecular labeling reagents^22^. This technique could potentially be useful for detecting drug concentrations and distributions within target tumors and organs, which have complicated structures. Furthermore, maturation and standardization of this procedure could also contribute to the prediction of efficacy and safety during the drug development process. Several studies have reported on the development of quantitative MSI methodologies^23^,^24^. One quantitative approach is to create a standard curve by determining known concentrations of a target drug in sections of control tissue, after which this curve is then compared to the signal intensity observed for adjacent dosed tissue sections^25^,^26^,^27^,^28^; however, some problems may occur with this technique including issues with choosing an appropriate control tissue, difficulties when trying to examine cases with heterogeneous tissues that have been determined to be the cause of differences in ion suppression, or difficulties in deciding what the concentrations may be in dried spot areas. To avoid these potential problems, in this study, we selected a quantitative method using LC-MS/MS of adjacent serial sections^23^,^29^ and combined this with the use of an internal standard that was sprayed within the matrix.

Recent studies have used MSI to analyze the activity of various compounds with regard to their pharmaceutical transit into the brain^16^,^30^,^31^,^32^. However, there have been few reports that have mentioned the reproducibility and quantitative capacity of targeting compounds. Even when a compound is a substrate of MDR1, which closely regulates intestinal absorption as well as brain distribution, there have been other cases reported, similar to our current study, where the brain transitivity differed from the blood concentrations found after an oral administration of a drug^13^,^14^,^15^. Generally, the drug transport and transit into the brain has been evaluated through the use of radioisotopes or LC-MS/MS analyses of homogenized brain tissue, precluding accurate determination of where the drug localized in the brain tissue, brain parenchyma, CSF, or brain blood. In addition, the calculation of drug concentrations in the CSF is currently a controversial subject^33^. Therefore, our current approach was able to precisely evaluate drug distribution at the microscopic level in wild-type and *Mdr1a/b* KO mice and could be a powerful tool in drug development.

While the resolution of the MSI images was a limitation of our current study, a conflict still exist between the analytical resolution and sensitivity in our MSI system^34^. There are recent reports that have described single cell imaging through the use of different MALDI-MSI system^35^,^36^. Although our data showed the positional relationship between alectinib and the brain blood vessels, further improvements in the specificity, sensitivity, and image resolution may give us further novel insights into PK research at a cellular level.

Another limitation of our study is that even though our results showed an association between the brain distribution of alectinib and the substrate property of MDR1, we did not specifically examine the efficacy of alectinib with regard to brain metastasis. The reason for this is that we did not use a tumor xenografted model. Since it has been reported that alectinib has exhibited promising activity in lung cancer patients with brain metastasis^37^, our data might support the speculation that the intact brain transitivity of drugs or the substrate property of MDR1 do not directly correlate with the drug penetration and treatment efficacy in brain metastatic tumors^17^,^38^. Interestingly, a recent study showed that even macromolecule such as antibody-drug was possible to localize at metastatic brain lesions of breast cancer by PET imaging of ^64^Cu-labeled trastuzumab^39^. Further translational research using brain metastatic samples and MSI of drugs might be able to resolve this controversial clinical question.

In conclusion, the spatial measurement of drug concentrations in target tissues and organs is crucial for predicting efficacy and safety during drug development and clinical application, and MSI technology is widely expected to contribute to this evaluation. It is hoped that present approach of using quantitative MSI and *Mdr1a/b* KO mice during the evaluation of brain drug distribution might be helpful in the determination of reproducible PK and pharmacodynamic studies of investigational new compounds.

## Methods

### Chemicals

Alectinib (Alecensa^®^) was purchased from Chugai Pharmaceutical Co., Ltd. (Tokyo, Japan). Erlotinib-d6 was purchased from Toronto Research Chemicals Inc. (Ontario, CA). α-Cyano-4-hydroxycinnamic acid (α-CHCA) was purchased from Sigma-Aldrich (St. Louis, MO, USA). Formic acid (FA), acetonitrile, methanol, ethanol, isopropanol, NaCl, CaCl_2_, KCl, Mayer’s hematoxylin solution, 1% eosin Y solution for HE staining were purchased from Wako Pure Chemical Industries Ltd. (Osaka, Japan). HPLC grade 0.1% FA was prepared with de-ionized water, which was purified using a Milli-Q purification system (Millipore, MA, USA). All other chemicals and reagents required for the LC-MS/MS were of analytical grade and used without further purifications.

### Animal experiments

Animal studies were carried out according to the Guideline for Animal Experiments, drawn up by the Committee for Animal Experimentation of National Cancer Center, which meet the ethical standards required by the law and the guidelines about experimental animals in Japan. All experimental animal protocols were approved by the Institutional Animal Ethics Committee (IAEC) of the National Cancer Center (Permit Number: T14-025). Animal treatment experiments were performed at K.A.C, Ltd. (Kyoto, Japan). For the *in vivo* studies on the PK and drug distribution in the brain, male FVB (6–8 weeks, wild-type) and *Mdr1a/b* KO mice^40^ (6–8 weeks) were purchased from Taconic Biosciences, Inc. (Oxnard, CA, USA). For the oral injections, alectinib was dissolved in a 0.5% (w/v) methyl cellulose 400 solution.

### Pharmacokinetic studies in mice

Alectinib was administered orally to the FVB and *Mdr1a/b* KO mice at doses of 4 and 20 mg/kg (n = 4). At 1, 1.5, 2 and 4 hours after the drug administration, brain, blood and cerebrospinal fluid (CSF) samples were collected from each mouse. Blood samples were collected in heparinized tubes, and centrifuged at 1500 × g at 4 °C for 10 min to obtain the plasma. After freezing the brains in liquid N_2_, they were stored at −80 °C until analyzed. Plasma and CSF were also stored at −80 °C until analyzed.

### Measurement of alectinib in plasma, CSF, and tissue sections by LC-MS/MS

#### Sample preparation

Alectinib was extracted from the plasma and CSF samples by a solid-phase extraction method that used Oasis HLB 96-well plates (10 mg sorbent per well, Waters Corporation, Milford, MA, USA). Alectinib calibration standards ranging from 5–1000 ng/mL (linearity range R^2^ = 0.9977, accuracy: ±9%) and 3.75–375 ng/mL (linearity range R^2^ = 0.999, accuracy: ±14%) (Supplemental Figure 6) were prepared from standard solutions with mouse pooled plasma (Sigma-Aldrich) and artificial CSF buffer^41^ (buffer: 147 mM NaCl, 2.3 mM CaCl_2_, and 4 mM KCl), respectively, and used during the measurements of the mouse plasma and CSF, respectively. Erlotinib-d6 was used the internal standard (100 ng/mL).

#### LC-MS/MS analysis

Chromatographic separation of the alectinib, the plasma internal standard, erlotinib-d6, CSF, and the tissue sections was performed using an XBridge C18 HPLC column (2.1 × 5.0 mm, 3.5 μm) maintained at 40 °C. Mobile phases A and B consisted of 0.1% FA aqueous solution and methanol containing 0.1% FA, respectively. Separation was performed using an isocratic elution (A:B = 1:1) at a flow rate of 0.2 mL/min with a Nexera X2 series (Shimadzu, Co., Kyoto, Japan). The column was equilibrated with the mobile phase for 10 min, with a run time of 6 min for each 2 μL injection volume.

Quantitation was conducted by selected reaction monitoring on a QTRAP4500 mass spectrometer (AB SCIEX, Framingham, MA, USA) with electrospray ionization in the positive mode. The optimized electrospray ionization parameters were as follows: ion source temperature, 700 °C; curtain gas, setting 30; nebulizing gas (GS1), setting 60; turbo-ionspray gas (GS2), setting 60; ionspray voltage, 4500 V; declustering potential, 121 V; collision energy, 35 V. The selected reaction monitoring transitions were m/z 483.2 to 396.0 for alectinib and m/z 400.0 to 338.9 for erlotinib-d6. The dwell time was 500 ms for each transition channel. All data were acquired and analyzed using the Analyst version 1.6.1 software (AB SCIEX).

### qMSI

#### Sectioning

qMSI requires at least three serial sections (8 μm). Tissue was sliced at −20 °C with a cryomicrotome (Leica CM 1950, Tokyo, Japan). Brain tissues were sliced in both the coronal and sagittal directions (Supplemental Figure 1). The first and third sections were used for the LC-MS/MS measurements of the amount of alectinib contained in the tissue, while the second section was used for MALDI-MSI. The MSI sections were placed on indium-tin-oxide coated glass slides (ITO glass, Matsunami Glass Ind., Ltd., Tokyo, Japan).

#### Extraction

For the alectinib extraction, after vortexing the first and third tissue sections in 300 μL of 50% methanol/water for 1 min, sections were centrifuged at 12,000 × g for 10 min at 4 °C and the supernatants were collected. A portion of the supernatant was used to measure the protein concentration using a Qubit 2.0 Fluorometer (Thermo Fisher Scientific, MA, USA) and was also used as a reference for the quantitation of alectinib. A standard addition method^42^ was used for quantitating alectinib in the samples to avoid the matrix effect. Alectinib in the pieces that were cut off by LMD (Leica LMD 6500, Tokyo, Japan) from parts of the three additive serial sections of the tissue was also extracted using the above procedure. The processed samples were then analyzed by LC-MS/MS.

#### MALDI-MSI

The two-step matrix technique was used^43^,^44^. α-CHCA was first coated on the slide using a homebuilt sublimation apparatus at 250 °C for 8 min, after which 10 mg/mL of α-CHCA solution containing 30% acetonitrile, 10% isopropanol, and 0.1% FA was sequentially sprayed onto the slide using a sprayer (PS270, GSI Creos Corp., Tokyo, Japan) according to the following 10 cycles: first three cycles of spraying for 3 seconds at 90-second intervals, next seven cycles of spraying for 1 second at 30-second intervals. For examination of the ionization efficiency of alectinib on the tissue section, erlotinib-d6 was used as the internal standard (IS) in the α-CHCA solution (2 μg/mL).

MALDI-MSI acquisition was performed using an iMScope (Shimadzu). This instrument consisted of an optical microscope and a quadrupole ion trap time-of-flight (qIT-TOF) analyzer with an atmospheric pressure MALDI source consisting of a 355-nm Nd:YAG laser (1,000 Hz, minimum spot diameter <10 μm)^34^. MSI data for the brain sections were acquired with a pixel step size for the surface raster set to a spatial resolution of 80 or 20 μm. A mass spectrum was obtained with 150 (50 × 3 times) laser shots each over a mass range of 50–500 Da at a resolution of 10,000 at m/z 1,000.

The optimal transitions for the alectinib and erlotinib-d6 as IS used for the MSI were m/z 483.3 to 396.2 (Supplemental Figure 5) and m/z 400.2 to 339.2, respectively. The IS data were acquired with 40 μm shift from the laser spot obtained with alectinib. Heme was measured as a surrogate marker of the vasculature by monitoring the ion of m/z 616.2 ± 0.02^30^, and the specificity of heme was confirmed by MS/MS analysis (m/z 616.2 to 557.2, data not shown).

Molecular images were visualized using the Imaging MS Solution ver. 1.11 (Shimadzu) with a scale bar of absolute ion intensity. Biomap 3.8.0.4 (Novartis Institutes for BioMedical Research, Basel, Switzerland) was also used to display the divided images by the IS or qMSI images, in which we converted the ion intensity of the scale bar into the amount of alectinib using the sum of intensity in MSI and the total quantity of alectinib using LC-MS/MS analysis of serial sections.

### Immunohistochemistry

Brain tissue sections were serially sectioned at an 8 μm thickness for immunohistochemical detection of vascular endothelial cells and MDR1. After fixing the sections with cold methanol at −20 °C for 10 min, sections were dried at ambient temperature, and then incubated with the primary anti-CD31 antibody (#ab28364, Abcam, Cambridge, MA, USA) at a 1:50 dilution or with anti-MDR1 (#ab170904, Abcam) at a 1:50 dilution overnight at 4 °C. After the incubation, sections were washed with 1 × TBS buffer and then further incubated with SignalStain Boost IHC Detection Reagent (anti-rabbit, Cell Signaling Technology, Tokyo, Japan) at room temperature for 30 minutes. BZ-X710 microscope (Keyence, Itasca, IL, USA) was used for the observation of stained sections and calculation of the section area.

### Statistical analysis

Unless stated otherwise, all data are presented as the mean ± standard deviation of the mean (SD) from three or more animals. All quantification and calibration data were statistically processed using JMP software version 12.0.1 for MAC (SAS Institute Japan, Tokyo, Japan). The significance of differences was evaluated using a unilateral *t*-test, with a *P* value < 0.05 considered to be statistically significant. Pharmacokinetic parameters for alectinib in the mouse plasma were calculated using the non-compartmental analysis of the WinNonlin software (version 6.3, Pharsight Corp., Mountain View, CA, USA).

## Additional Information

**How to cite this article**: Aikawa, H. *et al.* Visualizing spatial distribution of alectinib in murine brain using quantitative mass spectrometry imaging. *Sci. Rep.* **6**, 23749; doi: 10.1038/srep23749 (2016).

## Supplementary Material

Supplementary Information
