# Supplementary material for: Visualizing spatial distribution of alectinib in murine brain using quantitative mass spectrometry imaging
Source: Sci Rep. 2016 Mar 30;6:23749. doi: 10.1038/srep23749 (PMC4812395; doi:10.1038/srep23749)
Supplement: Supplementary Information [file srep23749-s1.pdf]

Visualizing spatial distribution of alectinib in murine brain using quantitative mass spectrometry imaging

Hiroaki Aikawa[1]*, Mitsuhiro Hayashi[1,2]*, Shoraku Ryu[2], Makiko Yamashita[2], Naoto Ohtsuka[3], Masanobu Nishidate[2,4,5], Yasuhiro Fujiwara[6], Akinobu Hamada[1,2,5]

Affiliation information

[1] Division of Clinical Pharmacology and Translational Research, Exploratory Oncology Research and Clinical Trial Center, National Cancer Center, 5-1-1 Tsukiji, Chuo-ku, Tokyo 104-0045, Japan

[2] Department of Molecular Imaging and Pharmacokinetics, National Cancer Center Research Institute, 5-1-1 Tsukiji, Chuo-ku, Tokyo 104-0045, Japan

[3] Shimadzu Techno-Research Inc., 3-19-2, Minamirokugo, Ohta-ku, Tokyo 144-0045, Japan

[4] Translational Clinical Research Science & Strategy Dept., Chugai Pharmaceutical Co., Ltd., 200 Kajiwara, Kamakura, Kanagawa 247-8530, Japan

[5]Department of Medical Oncology and Translational Research, Graduate school of Medical Sciences, Kumamoto University, 1-1-1 Honjo, Chuo-ku, Kumamoto 860-8556, Japan

[6] Strategic Planning Bureau, National Cancer Center, 5-1-1 Tsukiji, Chuo-ku, Tokyo 104-0045, Japan

Corresponding Author: Akinobu Hamada, PhD

Mailing address: Division of Clinical Pharmacology and Translational Research
Exploratory Oncology Research and Clinical Trial Center
National Cancer Center
5-1-1 Tsukiji, Chuo-ku, Tokyo 104-0045, Japan

Phone: +81-3-3542-2511 (EXT 4260) **FAX:** +81-3-3545-3567

E-mail: akhamad@ncc.go.jp

*These authors contributed equally to this study.

Supplemental Information

Supplemental Figures

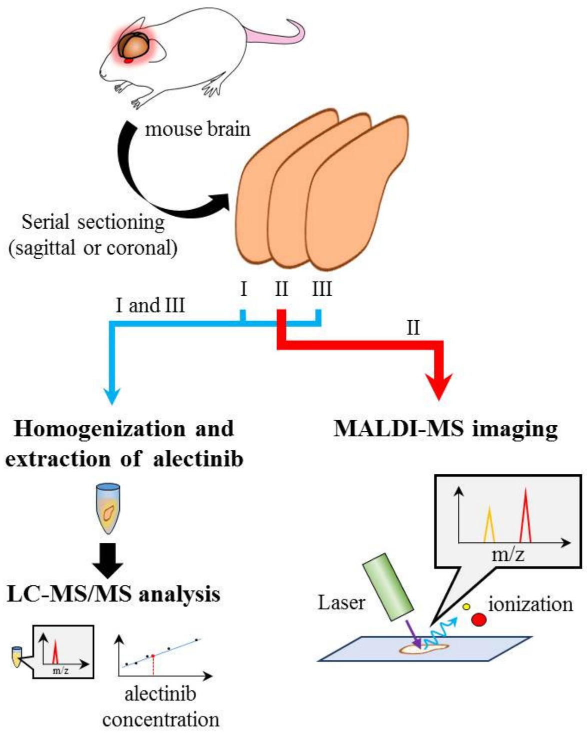

Supplemental Figure 1

Schematic of the quantitative MSI (qMSI) approach.

Three serial sections were used to build up the qMSI image. One section was used to detect the alectinib distribution by MALDI-MSI, whereas the other two were used to quantify the amount of alectinib. The signal intensity of the images detected by MALDI-MSI was converted into the absolute quantity of alectinib found in the serial section.

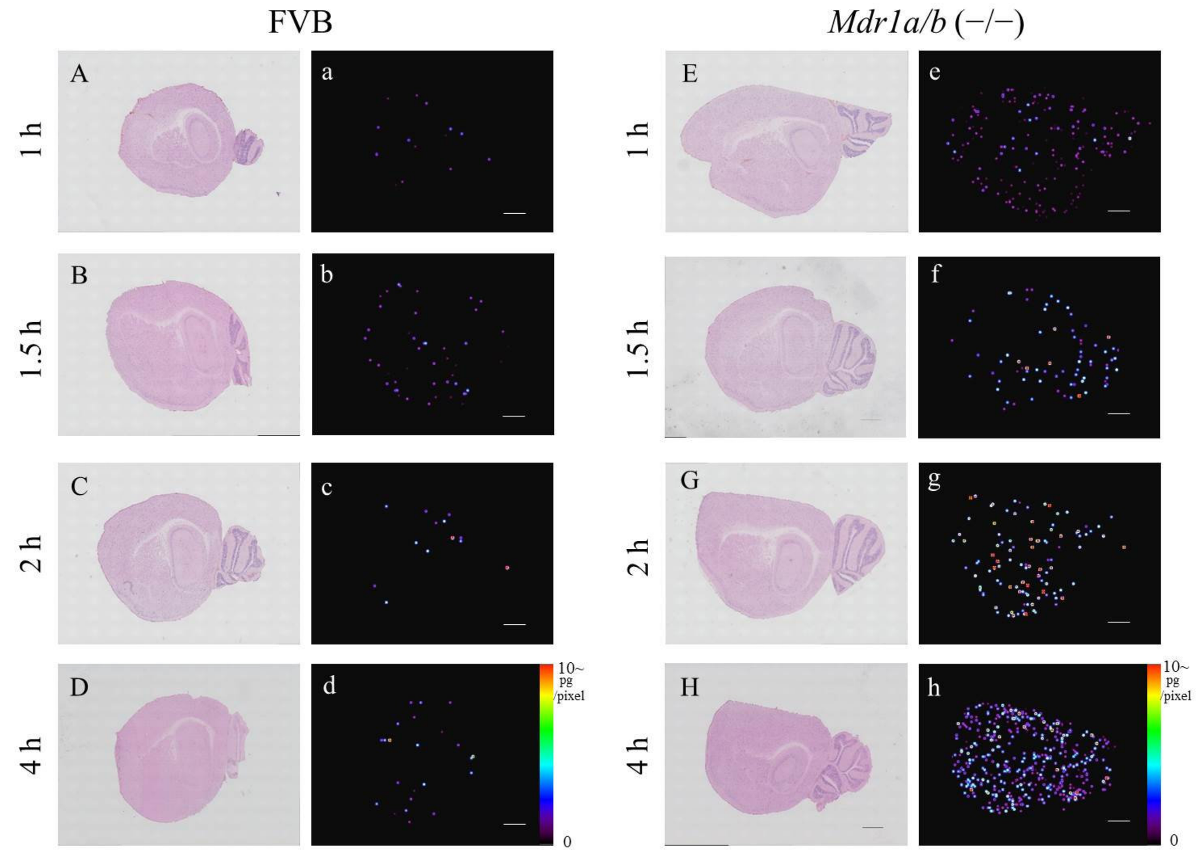

Supplemental Figure 2

Comparison of the qMSI images of alectinib at each time point after administration of 4 mg/kg.

(A-H) HE staining and (a-h) qMSI images of alectinib in brain tissue sections at 1, 1.5, 2, and 4 hours after oral administration of 4 mg/kg dose in FVB and *Mdr1a/b* (−/−) mice. MALDI-MSI was performed with a resolution of 80 µm. Scale bar: 1000 µm.

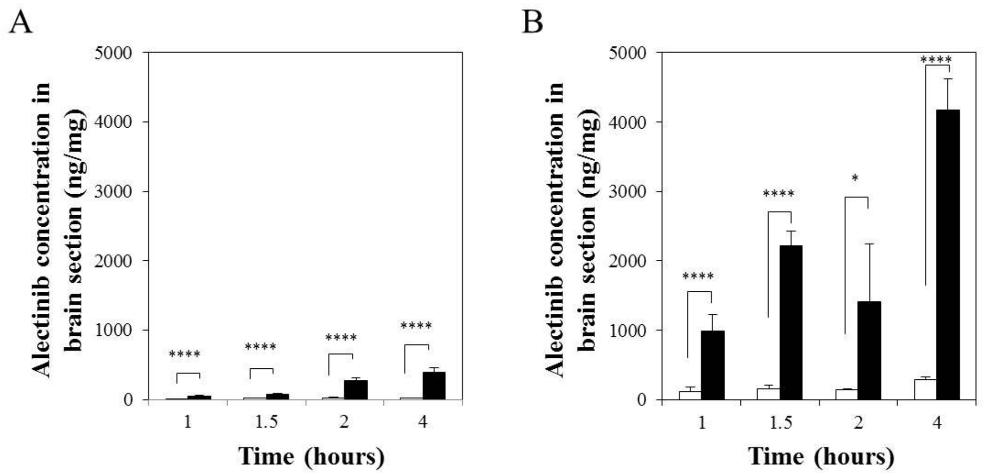

Supplemental Figure 3

Relative alectinib concentrations in brain tissue sections.

The total amounts of alectinib in brain tissue sections after a dose of 4 mg/kg (A) and 20 mg/kg (B) were measured by LC-MS/MS and adjusted for the protein concentration of the extraction solution from each tissue section (ng/mg). FVB: open bars, *Mdr1a/b* KO: filled bars. Data shown are the mean ± SD. n = 4. Statistically significant differences: *$P < 0.05$ and ****$P < 0.0005$.

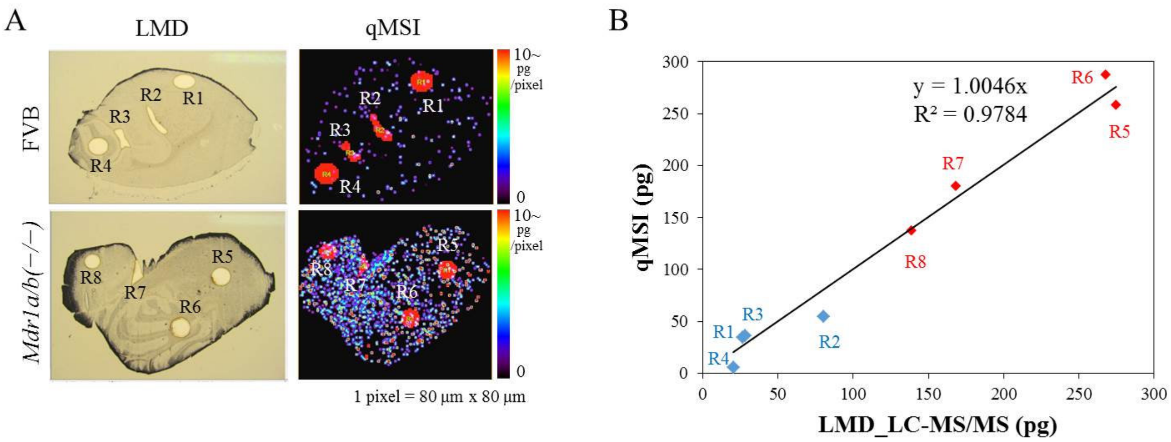

Supplemental Figure 4

Confirmation of the quantitative distribution of alectinib with qMSI by using laser microdissection.

(A) Mouse brain sections at 1 hour after administration of 20 mg/kg alectinib were used to confirm the quantitative alectinib distribution in the qMSI image (80 µm resolution) in FVB and *Mdr1a/b* KO mice. Laser microdissection was used to cut out eight separate regions (R1-R8) of the additional brain sections, and alectinib in those tissue regions was quantified by LC-MS/MS. (B) The correlation between the amounts of alectinib measured from dissected R1-R8 regions and those calculated from complementary R1-R8 regions of qMSI images was examined. FVB: blue, R1-R4; *Mdr1a/b* KO: red, R5-R8.

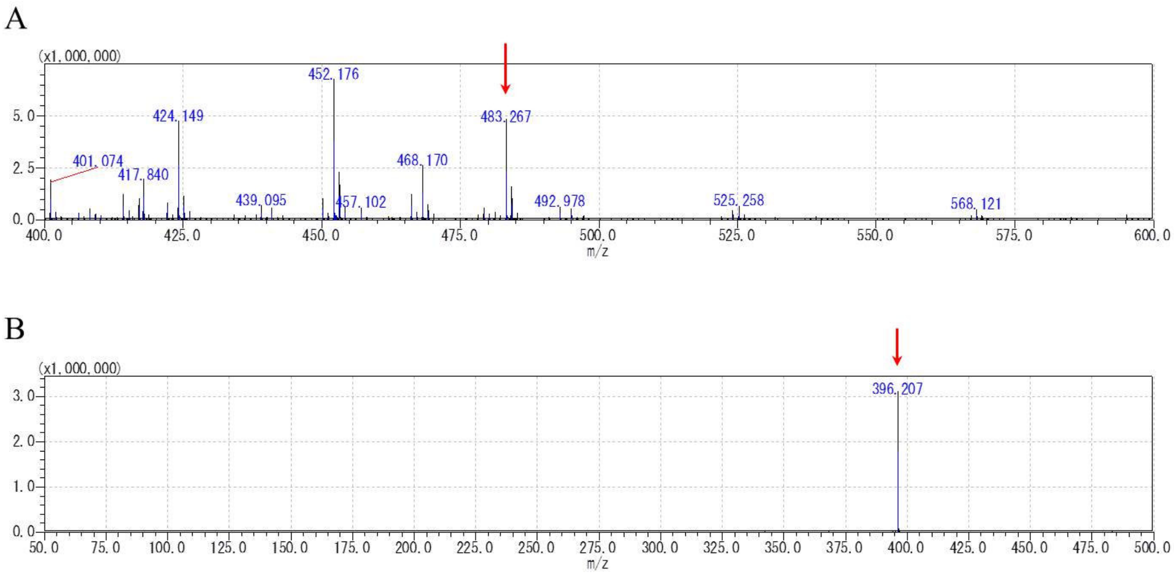

Supplemental Figure 5

MS/MS spectra of alectinib in MALDI-MSI analysis.

In this study, the MS/MS method in MALDI-MSI was used to detect alectinib to improve selectivity. Single MS spectra (A) and MS/MS (m/z 483.1 to 396.2) spectra (B) are shown for the analyses of the standard alectinib compound.

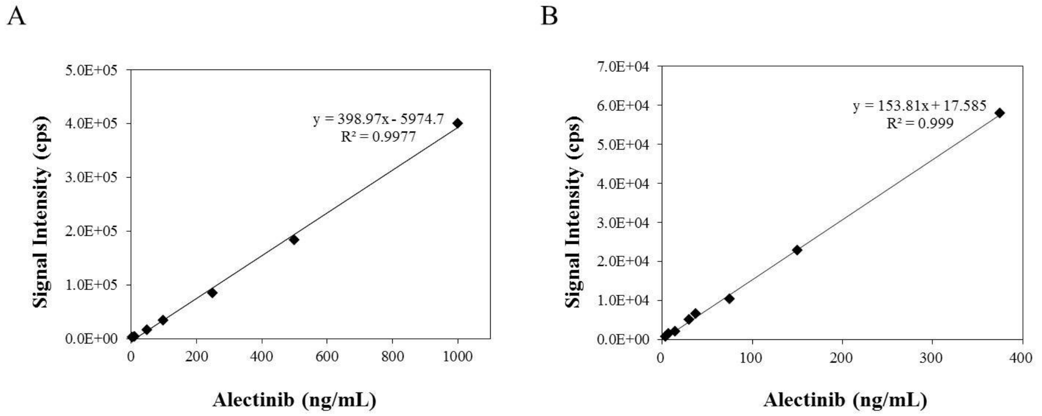

Supplemental Figure 6

Standard curve of alectinib for LC-MS/MS analyses of plasma and CSF samples.

The standard curves of alectinib for analysis of plasma (A) and CSF (B) by LC-MS/MS are shown. Alectinib calibration standards ranged from 5-1000 ng/mL (linearity range R^2 = 0.9977, accuracy: ±9%) in plasma and 3.75-375 ng/mL (linearity range R^2 = 0.999, accuracy: ±14%) in CSF. cps: count per second.

Supplemental Tables

Supplemental Table 1. Pharmacokinetics analysis of the plasma concentration of alectinib following oral administration in FVB and *Mdr1a/b* (−/−) mice.

	(hr)	lambda_z_conc (ng/mL)	AUC hr*ng/mL	AUMC hr*hr*ng/mL
4 mg/kg FVB	1	483.8	241.9	241.9
	1.5	990.5	610.4	734.3
	2	1136	1142	1674
	4	1100	3378	8345
4 mg/kg *Mdr1*(-/-)	1	694.8	347.4	347.4
	1.5	1145	807.3	950.4
	2	1015	1347	1887
	4	1243	3604	8886
20 mg/kg FVB	1	4163	2081	2081
	1.5	5863	4587	5320
	2	6460	7668	10748
	4	7120	21248	52149
20 mg/kg *Mdr1*(-/-)	1	4083	2041	2041
	1.5	5095	4336	4972
	2	5990	7107	9878
	4	7308	20404	51088

Supplemental Table 2. Alectinib concentrations in the cerebrospinal fluid of FVB and *Mdr1a/b* (−/−) mice.

		alectinib (ng/ml) in CSF[1]	
alectinib dose	hour	FVB	*Mdr1a/b* KO
4 mg/kg_1h		ND[2]	<LLOQ[3]
		ND	ND
		ND	ND
		ND*	5.093*
4 mg/kg_1.5h		ND*	ND
		ND	<LLOQ
		ND	<LLOQ
		<LLOQ	<LLOQ*
4 mg/kg_2h		ND	ND
		ND	ND
		<LLOQ	ND
		<LLOQ	<LLOQ*
4 mg/kg_4h		ND	ND
		ND	ND
		ND	ND
		<LLOQ	<LLOQ
20 mg/kg_1h		ND	<LLOQ
		<LLOQ	<LLOQ*
		5.03	5.54
		70.35*	44.93*
20 mg/kg_1.5h		ND	<LLOQ
		<LLOQ	<LLOQ
		5.74*	4.78
		58.35*	25.05*
20 mg/kg_2h		ND	<LLOQ
		<LLOQ	<LLOQ
		4.55	6.54
		14.33	13.65
20 mg/kg_4h		ND	<LLOQ
		ND	3.98
		<LLOQ	4.42
		23.85	5.59

Abbreviations: [1]CSF, cerebrospinal fluid; [2]ND, not detected; [3]LLOQ, low limit of quantitation.

<LLOQ indicates <3.75 ng/ml of alectinib concentration. * indicates the CSF sample was contaminated with blood.

Supplemental Table 3. Area of brain tissue sections used for LC-MS/MS analysis.

alectinib dose	$\text{Status}_{\text{hours}}$	Areas of tissue (mm^2)		
4 mg/kg	FVB_{1h}	35.04	±	7.40
	$\text{FVB}_{1.5h}$	32.05	±	5.21
	FVB_{2h}	35.43	±	3.42
	FVB_{4h}	37.43	±	5.00
	Mdr1a/b KO_{1h}	40.30	±	4.82
	Mdr1a/b $\text{KO}_{1.5h}$	40.85	±	5.11
	Mdr1a/b KO_{2h}	48.63	±	11.13
	Mdr1a/b KO_{4h}	41.86	±	7.33
20 mg/kg	FVB_{1h}	41.80	±	4.51
	$\text{FVB}_{1.5h}$	36.13	±	5.12
	FVB_{2h}	35.00	±	7.98
	FVB_{4h}	34.81	±	8.44
	Mdr1a/b KO_{1h}	36.83	±	3.28
	Mdr1a/b $\text{KO}_{1.5h}$	40.07	±	8.27
	Mdr1a/b KO_{2h}	41.14	±	6.10
	Mdr1a/b KO_{4h}	29.85	±	4.27

Mean ± SD. n = 4.

Supplemental Table 4. Alectinib concentration in mouse brain sections by LC-MS/MS.

	4 mg/kg dose		20 mg/kg dose	
	FVB	*Mdr1a/b* KO	FVB	*Mdr1a/b* KO
hours	alectinib, ng/mm^3	alectinib, ng/mm^3	alectinib, ng/mm^3	alectinib, ng/mm^3
1	0.15 ± 0.03	1.17 ± 0.22	3.32 ± 1.55	28.35 ± 7.91
1.5	0.41 ± 0.09	2.15 ± 0.39	4.93 ± 1.70	52.75 ± 6.09
2	0.73 ± 0.15	7.47 ± 2.13	4.29 ± 0.94	47.78 ± 30.97
4	0.53 ± 0.16	10.64 ± 1.58	8.24 ± 1.63	119.08 ± 2.31

Mean ± SD. n = 4.

Supplemental Table 5. Alectinib quantitation from both the measurement of LC-MS/MS for laser microdissected regions and the calculation from qMSI images for complementary regions.

Region	area (mm^2)	LMD[1] (pg)	qMSI[2] (pg)
$R1_{FVB}$	0.52	26.6	34.2
$R2_{FVB}$	0.34	80.3	54.6
$R3_{FVB}$	0.28	28.4	36.0
$R4_{FVB}$	0.49	20.3	5.6
R5 *Mdr1a/b* KO	0.50	267.8	287.7
R6 *Mdr1a/b* KO	0.44	274.6	258.6
R7 *Mdr1a/b* KO	0.23	168.1	180.5
R8 *Mdr1a/b* KO	0.42	138.6	137.6

Abbreviations: [1] LMD, laser micro dissection; [2] qMSI, quantitative mass spectrometry imaging. .
